# Supplementary material for: Hot spot prediction in protein-protein interactions by an ensemble system
Source: BMC Syst Biol. 2018 Dec 31;12(Suppl 9):132. doi: 10.1186/s12918-018-0665-8 (PMC6311905; doi:10.1186/s12918-018-0665-8)
Supplement: Supplementary file 2 — All relevant MATLAB code. A simple Matlab implement of our predictor. (ZIP 19,742 kb) [file 12918_2018_665_MOESM2_ESM.zip › Additional file 2/Read ASA/ASA website/dbMPIKT/1MHPH.html]

 NetSurfP - Protein Surface Accessibility and Secondary Structure Predictions

  

|  |  |  |
| --- | --- | --- |
|  |  | NetSurfP - Protein Surface Accessibility and Secondary Structure Predictions  Technical University of Denmark |

  


---

```
# For publication of results, please cite:
# A generic method for assignment of reliability scores applied to solvent accessibility predictions.
# Bent Petersen, Thomas Nordahl Petersen, Pernille Andersen, Morten Nielsen and Claus Lundegaard
# BMC Structural Biology 2009, 9:51 doi:10.1186/1472-6807-9-51
#
# Column 1: Class assignment - B for buried or E for Exposed - Threshold: 25% exposure, but not based on RSA
# Column 2: Amino acid
# Column 3: Sequence name
# Column 4: Amino acid number
# Column 5: Relative Surface Accessibility - RSA
# Column 6: Absolute Surface Accessibility
# Column 7: Z-fit score for RSA prediction
# Column 8: Probability for Alpha-Helix
# Column 9: Probability for Beta-strand
# Column 10: Probability for Coil
E E  1MHPH                  1    0.825 144.058   1.284   0.003   0.003   0.994
B V  1MHPH                  2    0.220  33.753  -0.336   0.004   0.616   0.381
E Q  1MHPH                  3    0.449  80.120   1.296   0.002   0.816   0.182
B L  1MHPH                  4    0.162  29.681   0.665   0.002   0.816   0.182
E V  1MHPH                  5    0.420  64.493   0.602   0.003   0.718   0.279
B E  1MHPH                  6    0.243  42.400   0.774   0.004   0.420   0.576
E S  1MHPH                  7    0.458  53.642  -0.592   0.004   0.138   0.858
E G  1MHPH                  8    0.505  39.720  -1.896   0.018   0.047   0.935
E G  1MHPH                  9    0.537  42.223  -2.367   0.018   0.047   0.935
E G  1MHPH                 10    0.517  40.719  -2.007   0.019   0.141   0.840
E L  1MHPH                 11    0.417  76.389  -0.950   0.022   0.359   0.619
E V  1MHPH                 12    0.293  45.019   0.557   0.021   0.451   0.528
E Q  1MHPH                 13    0.383  68.422   1.142   0.020   0.205   0.775
E P  1MHPH                 14    0.476  67.502  -0.654   0.018   0.047   0.935
E G  1MHPH                 15    0.603  47.480  -1.952   0.005   0.045   0.951
E G  1MHPH                 16    0.435  34.242  -0.563   0.004   0.138   0.858
E S  1MHPH                 17    0.435  51.017  -0.420   0.004   0.420   0.576
B L  1MHPH                 18    0.149  27.264   0.773   0.002   0.816   0.182
E R  1MHPH                 19    0.418  95.814   0.845   0.001   0.900   0.099
B L  1MHPH                 20    0.078  14.337   0.664   0.001   0.900   0.099
B S  1MHPH                 21    0.164  19.244   0.668   0.001   0.900   0.099
B C  1MHPH                 22    0.043   6.023   0.203   0.001   0.900   0.099
B A  1MHPH                 23    0.303  33.335   0.539   0.001   0.900   0.099
B A  1MHPH                 24    0.100  11.020  -0.251   0.002   0.816   0.182
B S  1MHPH                 25    0.179  21.026  -0.654   0.004   0.514   0.481
B G  1MHPH                 26    0.216  17.031  -1.889   0.004   0.197   0.799
B F  1MHPH                 27    0.252  50.617  -0.959   0.005   0.336   0.660
B T  1MHPH                 28    0.166  23.094  -1.508   0.004   0.514   0.481
B F  1MHPH                 29    0.128  25.770  -1.066   0.021   0.451   0.528
B S  1MHPH                 30    0.197  23.100  -1.525   0.020   0.205   0.775
B R  1MHPH                 31    0.304  69.570  -1.334   0.018   0.088   0.893
B Y  1MHPH                 32    0.115  24.661  -0.552   0.019   0.141   0.840
B T  1MHPH                 33    0.093  12.857  -0.173   0.022   0.359   0.619
B M  1MHPH                 34    0.029   5.863   0.195   0.021   0.756   0.223
B S  1MHPH                 35    0.050   5.860   0.200   0.021   0.756   0.223
B W  1MHPH                 36    0.036   8.610   0.455   0.018   0.846   0.136
B V  1MHPH                 37    0.039   5.979   0.231   0.018   0.846   0.136
B R  1MHPH                 38    0.119  27.136   0.426   0.021   0.756   0.223
B Q  1MHPH                 39    0.100  17.806   0.506   0.021   0.451   0.528
B A  1MHPH                 40    0.213  23.440   0.768   0.005   0.045   0.951
E P  1MHPH                 41    0.375  53.198   0.632   0.005   0.015   0.979
E G  1MHPH                 42    0.562  44.245  -1.720   0.018   0.019   0.964
E K  1MHPH                 43    0.413  84.892   0.524   0.018   0.047   0.935
E G  1MHPH                 44    0.454  35.753  -0.390   0.018   0.088   0.893
B L  1MHPH                 45    0.172  31.402   0.939   0.022   0.359   0.619
B E  1MHPH                 46    0.101  17.610  -0.295   0.003   0.718   0.279
B W  1MHPH                 47    0.052  12.434   0.363   0.001   0.900   0.099
B V  1MHPH                 48    0.029   4.488   0.581   0.001   0.959   0.040
B A  1MHPH                 49    0.019   2.083  -0.153   0.001   0.959   0.040
B T  1MHPH                 50    0.096  13.287   0.127   0.001   0.959   0.040
B I  1MHPH                 51    0.030   5.513   0.296   0.001   0.959   0.040
B S  1MHPH                 52    0.195  22.866   0.322   0.004   0.616   0.381
E G  1MHPH                 53    0.378  29.788  -1.203   0.004   0.197   0.799
E G  1MHPH                 54    0.571  44.938  -2.427   0.005   0.015   0.979
E G  1MHPH                 55    0.399  31.401  -2.357   0.018   0.019   0.964
E H  1MHPH                 56    0.483  87.803  -0.650   0.018   0.047   0.935
E T  1MHPH                 57    0.361  50.015  -0.482   0.019   0.141   0.840
E Y  1MHPH                 58    0.428  91.464   0.021   0.064   0.216   0.721
B Y  1MHPH                 59    0.219  46.736   0.262   0.056   0.142   0.802
B L  1MHPH                 60    0.237  43.431  -0.328   0.053   0.043   0.903
E D  1MHPH                 61    0.652  93.996  -0.641   0.053   0.043   0.903
E S  1MHPH                 62    0.399  46.739  -0.025   0.053   0.043   0.903
B V  1MHPH                 63    0.084  12.895  -0.284   0.018   0.088   0.893
E K  1MHPH                 64    0.394  81.025   0.706   0.019   0.141   0.840
B G  1MHPH                 65    0.210  16.551  -1.356   0.005   0.262   0.733
B R  1MHPH                 66    0.207  47.517   0.615   0.003   0.718   0.279
B F  1MHPH                 67    0.051  10.216  -0.356   0.001   0.900   0.099
B T  1MHPH                 68    0.113  15.631   0.363   0.001   0.959   0.040
B I  1MHPH                 69    0.045   8.306   0.557   0.001   0.959   0.040
B S  1MHPH                 70    0.087  10.208   0.528   0.002   0.816   0.182
E R  1MHPH                 71    0.255  58.418   0.749   0.004   0.616   0.381
B D  1MHPH                 72    0.239  34.498  -0.588   0.004   0.420   0.576
E N  1MHPH                 73    0.431  63.040  -0.251   0.018   0.088   0.893
B S  1MHPH                 74    0.244  28.632  -1.851   0.018   0.047   0.935
E K  1MHPH                 75    0.525 107.890   0.770   0.018   0.088   0.893
B N  1MHPH                 76    0.260  38.035  -0.363   0.022   0.359   0.619
B T  1MHPH                 77    0.203  28.114   0.907   0.018   0.846   0.136
B L  1MHPH                 78    0.076  13.861  -0.562   0.006   0.962   0.032
B Y  1MHPH                 79    0.174  37.162   0.344   0.006   0.962   0.032
B L  1MHPH                 80    0.047   8.532   0.699   0.006   0.962   0.032
B Q  1MHPH                 81    0.188  33.648  -0.010   0.011   0.918   0.071
B M  1MHPH                 82    0.078  15.508  -1.194   0.004   0.616   0.381
E N  1MHPH                 83    0.373  54.651  -0.220   0.005   0.336   0.660
E S  1MHPH                 84    0.404  47.302  -0.721   0.004   0.197   0.799
B L  1MHPH                 85    0.127  23.180  -0.183   0.004   0.085   0.910
E R  1MHPH                 86    0.506 115.851   1.095   0.018   0.088   0.893
E A  1MHPH                 87    0.385  42.405  -0.559   0.052   0.084   0.864
E E  1MHPH                 88    0.487  85.114  -0.626   0.053   0.043   0.903
B D  1MHPH                 89    0.220  31.673  -0.807   0.052   0.084   0.864
B T  1MHPH                 90    0.129  17.906   0.752   0.066   0.296   0.638
B A  1MHPH                 91    0.042   4.639  -0.249   0.021   0.756   0.223
B V  1MHPH                 92    0.079  12.112   0.614   0.018   0.846   0.136
B Y  1MHPH                 93    0.039   8.398   0.756   0.018   0.846   0.136
B Y  1MHPH                 94    0.052  11.134   0.816   0.021   0.756   0.223
B C  1MHPH                 95    0.019   2.682   1.238   0.023   0.655   0.322
B T  1MHPH                 96    0.028   3.925  -0.155   0.022   0.552   0.426
B R  1MHPH                 97    0.151  34.487   0.067   0.022   0.359   0.619
B G  1MHPH                 98    0.286  22.492  -0.780   0.020   0.205   0.775
B F  1MHPH                 99    0.291  58.424  -0.755   0.018   0.088   0.893
B G  1MHPH                100    0.300  23.610  -1.487   0.018   0.047   0.935
E D  1MHPH                101    0.463  66.776  -1.732   0.018   0.047   0.935
E G  1MHPH                102    0.352  27.702  -1.740   0.018   0.047   0.935
B G  1MHPH                103    0.263  20.730  -0.683   0.018   0.088   0.893
B Y  1MHPH                104    0.121  25.922  -0.560   0.019   0.141   0.840
B F  1MHPH                105    0.066  13.306  -0.061   0.021   0.279   0.699
B D  1MHPH                106    0.088  12.710  -0.898   0.021   0.279   0.699
B V  1MHPH                107    0.069  10.651  -0.669   0.021   0.279   0.699
B W  1MHPH                108    0.050  11.977  -0.830   0.020   0.205   0.775
B G  1MHPH                109    0.049   3.880  -1.670   0.004   0.138   0.858
B Q  1MHPH                110    0.179  31.880  -0.304   0.004   0.085   0.910
B G  1MHPH                111    0.110   8.665  -1.622   0.005   0.262   0.733
B T  1MHPH                112    0.066   9.113   0.164   0.004   0.616   0.381
E L  1MHPH                113    0.217  39.696   0.266   0.001   0.900   0.099
B V  1MHPH                114    0.034   5.195   0.288   0.001   0.900   0.099
B T  1MHPH                115    0.071   9.848   0.252   0.001   0.900   0.099
B V  1MHPH                116    0.044   6.809   0.078   0.002   0.816   0.182
B S  1MHPH                117    0.105  12.318   0.026   0.004   0.514   0.481
B S  1MHPH                118    0.138  16.138  -1.038   0.019   0.141   0.840
E A  1MHPH                119    0.314  34.559  -1.564   0.018   0.047   0.935
E S  1MHPH                120    0.372  43.552  -0.703   0.018   0.047   0.935
E T  1MHPH                121    0.309  42.914  -1.191   0.018   0.047   0.935
E K  1MHPH                122    0.582 119.800  -1.539   0.018   0.047   0.935
E G  1MHPH                123    0.520  40.948  -0.939   0.018   0.047   0.935
B P  1MHPH                124    0.311  44.131  -1.946   0.018   0.047   0.935
E S  1MHPH                125    0.458  53.724  -0.966   0.018   0.088   0.893
E V  1MHPH                126    0.464  71.317  -0.891   0.052   0.084   0.864
B F  1MHPH                127    0.250  50.215  -1.134   0.052   0.084   0.864
E P  1MHPH                128    0.482  68.396  -0.881   0.020   0.205   0.775
B L  1MHPH                129    0.262  47.936  -2.214   0.021   0.279   0.699
E A  1MHPH                130    0.360  39.639  -1.385   0.020   0.205   0.775
E P  1MHPH                131    0.351  49.764  -2.266   0.004   0.085   0.910
B S  1MHPH                132    0.257  30.074  -2.760   0.005   0.045   0.951
E S  1MHPH                133    0.481  56.338  -2.683   0.005   0.045   0.951
E K  1MHPH                134    0.636 130.805  -1.926   0.005   0.015   0.979
E S  1MHPH                135    0.329  38.512  -2.248   0.005   0.015   0.979
E T  1MHPH                136    0.441  61.139  -1.289   0.003   0.003   0.994
E S  1MHPH                137    0.540  63.288  -1.629   0.003   0.003   0.994
E G  1MHPH                138    0.673  52.926  -2.196   0.003   0.003   0.994
E G  1MHPH                139    0.474  37.327  -1.751   0.005   0.045   0.951
E T  1MHPH                140    0.422  58.573   0.587   0.004   0.420   0.576
B A  1MHPH                141    0.076   8.386   0.744   0.001   0.900   0.099
B A  1MHPH                142    0.194  21.423   0.797   0.001   0.959   0.040
B L  1MHPH                143    0.043   7.910   0.244   0.001   0.959   0.040
B G  1MHPH                144    0.048   3.801  -0.028   0.001   0.900   0.099
B C  1MHPH                145    0.022   3.019   0.829   0.001   0.900   0.099
B L  1MHPH                146    0.140  25.707   0.666   0.001   0.959   0.040
B V  1MHPH                147    0.057   8.699  -0.002   0.002   0.816   0.182
B K  1MHPH                148    0.250  51.384   0.115   0.004   0.514   0.481
E D  1MHPH                149    0.418  60.291  -1.800   0.004   0.197   0.799
B Y  1MHPH                150    0.250  53.318  -0.918   0.004   0.197   0.799
E F  1MHPH                151    0.310  62.137  -0.485   0.004   0.197   0.799
B P  1MHPH                152    0.234  33.261  -1.383   0.004   0.138   0.858
E E  1MHPH                153    0.587 102.479  -1.107   0.004   0.138   0.858
E P  1MHPH                154    0.425  60.279  -1.280   0.004   0.197   0.799
B V  1MHPH                155    0.162  24.946   0.045   0.003   0.718   0.279
E T  1MHPH                156    0.341  47.255  -0.425   0.001   0.900   0.099
B V  1MHPH                157    0.067  10.236   0.226   0.001   0.900   0.099
B S  1MHPH                158    0.283  33.132   0.166   0.001   0.900   0.099
B W  1MHPH                159    0.087  20.827  -0.207   0.011   0.918   0.071
E N  1MHPH                160    0.328  48.063   0.004   0.003   0.718   0.279
E S  1MHPH                161    0.480  56.291  -0.807   0.005   0.336   0.660
E G  1MHPH                162    0.517  40.649  -0.988   0.018   0.088   0.893
E A  1MHPH                163    0.533  58.737  -0.229   0.018   0.019   0.964
E L  1MHPH                164    0.504  92.264  -0.932   0.018   0.047   0.935
E T  1MHPH                165    0.604  83.719  -2.106   0.018   0.047   0.935
E S  1MHPH                166    0.523  61.272  -0.361   0.005   0.045   0.951
E G  1MHPH                167    0.468  36.792  -0.462   0.004   0.138   0.858
B V  1MHPH                168    0.162  24.976  -0.377   0.004   0.420   0.576
E H  1MHPH                169    0.347  63.101   0.402   0.002   0.816   0.182
B T  1MHPH                170    0.264  36.561  -0.211   0.001   0.900   0.099
B F  1MHPH                171    0.176  35.263   1.049   0.002   0.816   0.182
B P  1MHPH                172    0.175  24.818  -0.745   0.022   0.552   0.426
B A  1MHPH                173    0.242  26.690  -1.044   0.003   0.718   0.279
B V  1MHPH                174    0.184  28.312  -1.853   0.001   0.900   0.099
E L  1MHPH                175    0.304  55.681  -0.365   0.003   0.718   0.279
E Q  1MHPH                176    0.434  77.441  -0.211   0.004   0.420   0.576
E S  1MHPH                177    0.720  84.361  -1.255   0.004   0.085   0.910
E S  1MHPH                178    0.764  89.588  -1.221   0.005   0.015   0.979
E G  1MHPH                179    0.457  35.990  -1.316   0.004   0.085   0.910
E L  1MHPH                180    0.386  70.640   0.819   0.003   0.718   0.279
B Y  1MHPH                181    0.144  30.687   0.318   0.001   0.900   0.099
B S  1MHPH                182    0.198  23.206  -0.563   0.001   0.900   0.099
B L  1MHPH                183    0.127  23.162  -0.875   0.001   0.959   0.040
B S  1MHPH                184    0.240  28.116  -0.058   0.001   0.959   0.040
B S  1MHPH                185    0.052   6.094  -0.658   0.001   0.959   0.040
B V  1MHPH                186    0.234  36.027   0.454   0.001   0.959   0.040
B V  1MHPH                187    0.061   9.345   0.395   0.001   0.959   0.040
E T  1MHPH                188    0.288  39.918   0.978   0.001   0.900   0.099
B V  1MHPH                189    0.070  10.774   1.013   0.001   0.900   0.099
E P  1MHPH                190    0.354  50.261   1.275   0.023   0.655   0.322
E S  1MHPH                191    0.401  47.044  -0.161   0.021   0.279   0.699
E S  1MHPH                192    0.626  73.391  -0.690   0.022   0.359   0.619
E S  1MHPH                193    0.606  71.047   0.491   0.021   0.279   0.699
B L  1MHPH                194    0.154  28.142  -0.650   0.004   0.138   0.858
E G  1MHPH                195    0.746  58.718   0.712   0.005   0.015   0.979
E T  1MHPH                196    0.764 105.967   0.974   0.005   0.015   0.979
E Q  1MHPH                197    0.564 100.641   0.675   0.005   0.262   0.733
E T  1MHPH                198    0.508  70.515   1.139   0.002   0.816   0.182
B Y  1MHPH                199    0.077  16.412   0.534   0.001   0.959   0.040
E I  1MHPH                200    0.379  70.078   1.828   0.001   0.959   0.040
B C  1MHPH                201    0.028   3.889  -0.199   0.001   0.959   0.040
E N  1MHPH                202    0.386  56.452   0.977   0.001   0.900   0.099
B V  1MHPH                203    0.082  12.557  -0.687   0.002   0.816   0.182
E N  1MHPH                204    0.566  82.906   0.576   0.002   0.816   0.182
B H  1MHPH                205    0.182  33.069   0.207   0.004   0.514   0.481
E K  1MHPH                206    0.635 130.558   0.334   0.005   0.015   0.979
E P  1MHPH                207    0.765 108.582  -0.979   0.005   0.015   0.979
E S  1MHPH                208    0.514  60.253  -0.263   0.005   0.045   0.951
E N  1MHPH                209    0.623  91.163   0.187   0.004   0.085   0.910
E T  1MHPH                210    0.510  70.765   0.823   0.005   0.262   0.733
E K  1MHPH                211    0.556 114.369   1.649   0.004   0.616   0.381
B V  1MHPH                212    0.089  13.664   0.097   0.002   0.816   0.182
E D  1MHPH                213    0.700 100.913   0.531   0.001   0.900   0.099
E K  1MHPH                214    0.311  63.973   1.079   0.001   0.900   0.099
E K  1MHPH                215    0.624 128.460   0.942   0.003   0.718   0.279
B V  1MHPH                216    0.209  32.108   0.474   0.005   0.336   0.660
E E  1MHPH                217    0.558  97.518   0.476   0.004   0.085   0.910
E P  1MHPH                218    0.517  73.376  -1.547   0.005   0.015   0.979
E K  1MHPH                219    0.864 177.807   0.245   0.000   0.000   0.999

Explain the output.  Go back.
```

---
